# In-hospital mortality after stomach cancer surgery in Spain and relationship with hospital volume of interventions

**DOI:** 10.1186/1471-2458-9-312

**Published:** 2009-08-27

**Authors:** Marisa Baré, Joan Cabrol, Jordi Real, Gemma Navarro, Rafel Campo, Carles Pericay, Antonio Sarría

**Affiliations:** 1 Cancer Screening Office/Epidemiology, UDIAT-Diagnostic Centre, Corporació Sanitària Parc Taulí-Institut Universitari (UAB), Parc Taulí s/n, Sabadell, Spain; 2 Department of General and Digestive Surgery, Corporació Sanitària Parc Taulí-Institut Universitari (UAB), Sabadell, Spain; 3 Epidemiology, Corporació Sanitària Parc Taulí-Institut Universitari (UAB), Sabadell, Spain; 4 Digestive Disease Department, Corporació Sanitària Parc Taulí-Institut Universitari (UAB), Sabadell, Spain; 5 Oncology Department, Corporació Sanitària Parc Taulí-Institut Universitari (UAB), Sabadell, Spain; 6 Agency for the Evaluation of Healthcare Technologies – AETS, Instituto de Salud Carlos III, Ministry of Health and Consumer Affairs, Madrid, Spain; 7 USR-Lleida. ICS-IDIAP. Lleida, Spain

## Abstract

**Background:**

There is no consensus about the possible relation between in-hospital mortality in surgery for gastric cancer and the hospital annual volume of interventions. The objectives were to identify factors associated to greater in-hospital mortality for surgery in gastric cancer and to analyze the possible independent relation between hospital annual volume and in-hospital mortality.

**Methods:**

We performed a retrospective cohort study of all patients discharged after surgery for stomach cancer during 2001–2002 in four regions of Spain using the Minimum Basic Data Set for Hospital Discharges. The overall and specific in-hospital mortality rates were estimated according to patient and hospital characteristics. We adjusted a logistic regression model in order to calculate the in-hospital mortality according to hospital volume.

**Results:**

There were 3241 discharges in 144 hospitals. In-hospital mortality was 10.3% (95% CI 9.3–11.4). A statistically significant relation was observed among age, type of admission, volume, and mortality, as well as diverse secondary diagnoses or the type of intervention. Hospital annual volume was associated to Charlson score, type of admission, region, length of stay and number of secondary diagnoses registered at discharge. In the adjusted model, increased age and urgent admission were associated to increased in-hospital mortality. Likewise, partial gastrectomy (Billroth I and II) and simple excision of lymphatic structure were associated with a lower probability of in-hospital mortality. No independent association was found between hospital volume and in-hospital mortality

**Conclusion:**

Despite the limitations of our study, our results corroborate the existence of patient, clinical, and intervention factors associated to greater hospital mortality, although we found no clear association between the volume of cases treated at a centre and hospital mortality.

## Background

### Importance of gastric cancer

Stomach cancer is the second most common malignancy of the digestive tract in developed countries [1]. In Spain, the incidence adjusted to the worldwide population ranges from 12.2 to 21.6 cases per 100 000 men, depending on the region; the incidence in women is slightly less than half that of men. Surgery and chemotherapy are the mainstays of treatment. However, surgery is associated with considerable morbidity and lesser though significant mortality. The few studies published on morbidity and mortality after surgery for gastric cancer report variable rates [2-4].

In Spain, gastric cancer surgery is performed in many types of hospitals and in all regions. On the other hand, there is no specific register that facilitates the assessment of process and outcomes of surgical interventions.

### Outcomes study and in-hospital mortality

In-hospital mortality has often been considered an outcome indicator directly related with the quality of care [5]. Because in-hospital mortality is an objective measurement that is readily available in hospital databases, it has been used to analyze and compare outcomes among different centres. However, to ensure valid comparison, it is necessary to adjust the rates by taking patients' baseline risk or comorbidities into account [6,7]; thus, different methods have been validated to be used with administrative databases with codes for diagnoses and procedures [8,9]. In the absence of specific registers, administrative databases are the main alternative for this kind of evaluation.

### Factors associated to in-hospital mortality in gastric cancer

In addition to patients' baseline condition, aspects related to the structure of the hospital, the experience of the professionals involved, and the surgical procedure itself can affect surgical outcomes. Likewise, a centre's volume of activity for a given type of surgical procedure, especially for cardiovascular and oncological interventions, has also been reported to affect post-operative mortality in several studies [10-14]. However, some recent studies question the relationship between volume of activity and outcome; the authors of these studies point out that even if increased volume of activity were responsible for better outcome, the mechanisms underlying improved outcomes are not clear [13,15,16]. On the other hand, different definitions and cut-off points referring to hospital volume could be responsible for the divergent results found among different studies.

### Study justification

Given the relatively high rate of in-hospital mortality for gastric cancer reported by various authors, the scarcity of studies that analyze the surgical outcomes of this malignancy in Spain, and the controversies related to the possible association between volume of activity and outcomes, this study aimed to: 1. estimate the in-hospital mortality in surgery for gastric cancer in different regions in Spain; 2. identify factors associated to greater in-hospital mortality; 3. analyze the possible relation between volume and in-hospital mortality.

## Methods

### Design, setting, patients, and source of information

We performed a retrospective cohort study (based on administrative database) of all patients discharged after surgery for stomach cancer during 2001 and 2002 in four regions of Spain. These regions represent about 52% of the total population. In Spain, there is neither a common oncological surgical registry nor a National Cancer Registry. For many years, all hospital discharges are homogenously recorded and centralized at the Department of Health in each of the 17 Autonomous Communities or regions in the administrative database called Minimum Basic Data Set for Hospital Discharges (MBDS-HD). This database contains the following information: date of birth, gender (male or female), type of admission (urgent or scheduled), destination on discharge (dead or alive), International Classification of Diseases 9^th ^revision Clinical Modification (ICD9CM) [17] codes for the main and secondary diagnoses, ICD codes for the main and secondary procedures performed, date of admission, and date of discharge.

We included all discharges corresponding to patients with a principal diagnosis of stomach cancer (ICD code: 151.XX) that had undergone total or partial gastrectomy (ICD code: 43.5–43.9).

### Groundwork with experts: proposing factors

Secondary diagnoses were grouped into 259 mutually exclusive categories using the Clinical Classifications Software (CCS) [18] developed by the Center for Organization and Delivery Studies in the Healthcare Cost and Utilization Project (HCUP) at the Agency for Healthcare Research and Quality (AHRQ).

To pre-select factors that might be associated to in-hospital mortality, we contacted oncologists, gastroenterologists, and surgeons from different centres. We asked them to propose a list of surgical factors, patient comorbidities, factors related to the severity of disease, and complications that they considered might increase the probability of in-hospital death during or after surgery. The possible factors suggested and corresponding ICD codes are listed in Appendix 1. Although the stage of the tumour was among the factors proposed, it was not included in the study because the MBDS-HD does not include a specific code for this factor and no population cancer registry was available.

The study was approved by the institutional review board of the Corporació Sanitària del Parc Taulí.

### Variables analyzed

Apart from the factors listed in the appendix, the following variables were considered: age group (≤50, 51–64, 65–74, 75–84, ≥ 85), gender, region, type of admission as recorded in the MBDS-HD (urgent or elective), and volume of discharges analyzed for each hospital. For each admission, the Charlson score was calculated from the codes for the secondary diagnoses using the Deyo [8] adaptation; each case was then grouped into one of four categories (0, 1, 2, > 2). We calculated the length of stay for each admission. We also created the variable 'number of secondary diagnosis coded' for each discharge, which was later recoded into the categories ≤ 3, 4–5, and ≥ 6.

### Definition of in-hospital mortality and hospital volume

In-hospital mortality was defined as death occurring during the hospital stay. The annual volume of discharges was defined as the mean number of discharges included in the study at a given centre per year. Annual volume of discharges was grouped into three categories according to terciles (<18, 18–35, >35) and into 7 volume categories corresponding to smaller ranges consisting of 10 discharges each.

### Statistical analysis

The unit of analysis was the hospital discharge. We carried out a descriptive analysis of all variables of interest. The overall and specific in-hospital mortality rates for stomach cancer were estimated as a function of the admission type, age group, gender, region, annual volume of discharges, CCS diagnoses selected, and type of surgical procedure. The 95% confidence intervals were calculated for the overall rate according to the normal approximation. The chi-square or the Fisher's exact test was used to determine whether the factors studied were associated to mortality. Then, the same type of analysis was used to compare some variables of interest (age, gender, mortality, Charlson score, type of admission, region), as a function of the 3 annual volume categories. We used the Kruskal-Wallis test to compare the mean number of secondary diagnoses registered per discharge and the mean length of stay.

Then, a logistic regression model was constructed to determine whether the different demographic (age, region), admission factors (urgent, number of secondary diagnoses), or comorbidities studied (Charlson, congestive heart failure, pancreatic disorders, cardiac dysrhythmias, nutritional deficiencies, gastrointestinal haemorrhage, other gastrointestinal disorders, invasion of other structures) were independently associated to the adjusted mortality. Only those secondary diagnoses considered comorbidities by the experts and not included in the Charlson score were considered for the model, so possible complications occurring as a consequence of the intervention were not included (see appendix 1). First, we selected variables present in more than 1% of cases (more than 30 cases) that had *p *values < 0.1 in the univariate analysis. Next, we used the forward conditional stepwise method to construct the model. The odds ratios and 95% confidence intervals were calculated. Finally, goodness of fit was evaluated by the Hosmer-Lemeshow X^2 ^statistic [19] and the area under the receiver operator characteristic (ROC) curve was calculated to assess the discriminative capacity of the model. Values ranging from 0.7 to 0.8 represent reasonable discrimination and values exceeding 0.8 represent good discrimination [20].

We evaluated the association between hospital volume and adjusted mortality by introducing the variable annual hospital volume (3 categories) in the logistic regression model and estimating its odds ratios and 95% confidence intervals.

We considered *p *< 0.05 significant for all tests. The SPSS 15.0 statistical package was used for all analyses.

## Results

During 2001 and 2002, there were 3241 discharges of patients operated on for stomach cancer in the four regions analyzed. Nearly two thirds of the discharges corresponded to men and the predominant age group was 65–75 years old (see table 1).

**Table 1:** Hospital mortality according to socio-demographic and admission variables.

	**Patients**		**In-hospital mortality**		
	**n**	**Col %**	**n**	**Row %**	**p-value**

**Gender**					
Male	2055	63.4	220	10.7	0.32
Female	1186	36.6	114	9.6	
**Age group**					
≤ 50	331	10.2	6	1.8	<0.01
51–64	770	23.8	48	6.2	
65–74	1093	33.7	100	9.1	
75–84	894	27.6	142	15.9	
≥ 85	153	4.7	38	24.8	
**Region**					
A^1^	420	13.0	44	10.5	0.10
B	1249	38.5	113	9.0	
C	1058	32.6	128	12.1	
D	514	15.9	49	9.5	
**Admission type**					
Urgent	970	29.9	147	15.2	<0.01
Elective	2271	70.1	187	8.2	
**Hospital volume**					
<18	1145	35.3	90	7.9	0.003
18–35	1050	32.4	123	11.7	
>35	1046	32.3	121	11.6	
**Charlson score**					
0	1576	48.6	153	9.7	0.05
1	516	15.9	55	10.7	
2	118	3.6	21	17.8	
≥ 3	1031	31.8	105	10.2	

^1 ^Only 2001 data

Median hospital stay (LOS) was 19 days (mean 25 (18); range 1–291 in the 144 hospitals included, and it was higher for urgent admissions than for elective ones (median 29 vs 15, p < 0.001). Crude in-hospital mortality was 10.3% (95% CI 9.3–11.4). No statistically significant differences in mortality were observed between regions (see table 1). A statistically significant relation was observed among age, type of admission, volume, and mortality. Statistically significant associations were found between mortality and several clinical factors, such as respiratory or renal failure, electrolyte disorders, acute myocardial infarction, peritonitis and intestinal abscess, congestive heart failure (CHF), cardiac dysrhythmia, gastrointestinal haemorrhage, or diverse complications of surgical procedures (tables 2 and 3). Mortality was significantly higher in tumours located in the *fundus *or *cardia *of stomach (p = 0.001). A trend toward higher mortality with higher volume was observed only in *fundus *or *cardia *tumours. Mortality was significantly lower in partial gastrectomy with anastomosis to the duodenum (Billroth I), and in simple or even in radical excision of lymphatic structures (lymphadenectomy) than in other surgical procedures, but only in locations other than the *cardia *or *fundus*.

**Table 2:** Hospital mortality according to clinical factors.

			**Patients**	**In-hospital mortality**		
			**N**	**n**	**Row %**	**p-value**

**Secondary diagnosis**						
Respiratory failure, insufficiency, arrest (adult)		No	3071	227	7.4	<0.01
		Yes	170	107	62.9	
Renal failure		No	3159	285	9.0	<0.01
		Yes	82	49	59.8	
Fluid and electrolyte disorders		No	3209	316	9.8	<0.01
		Yes	32	18	56.3	
Acute myocardial infarction		No	3234	330	10.2	<0.01
		Yes	7	4	57.1	
Peritonitis and intestinal abscess		No	3123	282	9.0	<0.01
		Yes	118	52	44.1	
Congestive heart failure, non-hypertensive		No	3173	310	9.8	<0.01
		Yes	68	24	35.3	
Pancreatic disorders (not diabetes)		No	3213	326	10.1	<0.01
		Yes	28	8	28.6	
Pneumonia		No	3131	304	9.7	<0.01
		Yes	110	30	27.3	
Cardiac dysrhythmias		No	3047	286	9.4	<0.01
		Yes	194	48	24.7	
Nutritional deficiencies		No	3215	328	10.2	0.03
		Yes	26	6	23.1	
Complications of surgical procedures or medical care		No	2302	121	5.3	<0.01
		Yes	939	213	22.7	
Gastrointestinal haemorrhage		No	3079	303	9.8	<0.01
		Yes	162	31	19.1	
Intestinal obstruction without hernia		No	3192	325	10.2	0.06
		Yes	49	9	18.4	
Other gastrointestinal disorders		No	3097	309	10.0	<0.01
		Yes	144	25	17.4	
Diabetes mellitus with complications		No	3215	330	10.3	0.39
		Yes	26	4	15.4	
Invasion of others structures		No	2840	273	9.6	<0.01
		Yes	401	61	15.2	
Phlebitis, thrombophlebitis, and thromboembolism		No	3190	328	10.3	0.73
		Yes	51	6	11.8	
Hypertension		No	2576	273	10.6	0.28
		Yes	665	61	9.2	
Urinary tract infections		No	3159	334	10.6	<0.01
		Yes	82	0		
Diverticulosis and diverticulitis		No	3184	334	10.5	0.01
		Yes	57	0		
**Anatomic localization of the tumour**	**volume**					
***Cardia/Fundus***	<18		106	12	11.3	0.14
	18–35		99	14	14.1	
	>35		115	21	18.3	
**Other/unspecified**	<18		1039	78	7.5	0.01
	18–35		951	109	11.5	
	>35		931	100	10.7	

**Table 3:** Hospital mortality according to surgical procedure.

			**Patients**	**In-hospital mortality**		
			**N**	**n**	**Row %**	**p-value**

**Procedures by anatomic localization**						
***Cardia/Fundus***	Regional lymph node excision	No	302	46	15.2	0.49
		Yes	18	1	5.6	
	Radical excision of other lymph nodes	No	299	47	15.7	0.05
		Yes	21	0		
	Simple excision of lymphatic structure	No	308	47	15.3	0.23
		Yes	12	0		
	Partial gastrectomy with anastomosis to oesophagus (proximal)		18	3	20.0	0.51
	Other partial gastrectomy		27	1	3.7	
	Total gastrectomy		250	38	15.2	
	Partial gastrectomy with anastomosis to jejunum (Billroth II)		20	4	20.0	
	Partial gastrectomy with anastomosis to duodenum (Billroth I)		5	1	20.0	
**Other/Unspecified**						
	Regional lymph node excision	No	2805	278	9.9	0.44
		Yes	116	9	7.8	
	Radical excision of other lymph nodes	No	2732	277	10.1	0.03
		Yes	189	10	5.3	
	Simple excision of lymphatic structure	No	2791	284	10.2	<0.01
		Yes	130	3	2.3	
	Partial gastrectomy with anastomosis to oesophagus (proximal)		7	2	28.6	0.01
	Other partial gastrectomy		590	72	12.2	
	Total gastrectomy		1096	111	10.1	
	Partial gastrectomy with anastomosis to jejunum (Billroth II)		984	89	9.0	
	Partial gastrectomy with anastomosis to duodenum (Billroth I)		244	13	5.3	

The Charlson index, the type of admission, the region, the number of secondary diagnosis registered, and the LOS were significantly associated to annual volume (Table 4). Thus, we found a greater proportion of patients with Charlson scores greater than or equal to 3 in hospitals performing more interventions compared to those performing fewer interventions. The proportion of urgent admissions and the LOS also increased with higher volume of interventions. Likewise, the higher the annual volume of interventions, the higher the number of secondary diagnoses recorded. Finally, hospital mortality was also significantly lower in the hospitals with lower volume of interventions.

**Table 4:** Patient or admission factors according to annual hospital volume.

		**Hospital volume**						
		
		**<18**	**Col %**	**18 – 35**	**Col %**	**>35**	**Col %**	**p-value**
**In-hospital mortality**	Yes	90	7.9	123	11.7	121	11.6	0.003
	No	1055	92.1	927	88.3	925	88.4	
								
**Gender**	Male	731	63.8	670	63.8	654	62.5	0.772
	Female	414	36.2	380	36.2	392	37.5	
								
**Age group**	≤ 50	128	11.2	108	10.3	95	9.1	
	51–65	270	23.6	249	23.7	251	24.0	
	65–75	386	33.7	344	32.8	363	34.7	0.778
	75–84	314	27.4	293	27.9	287	27.4	
	≥ 85	47	4.1	56	5.3	50	4.8	
								
**Charlson score**	0	662	57.8	482	45.9	432	41.3	
	1	173	15.1	164	15.6	179	17.1	0.000
	2	35	3.1	32	3.0	51	4.9	
	≥ 3	275	24.0	372	35.4	384	36.7	
								
**Admission type**	Urgent	265	23.1	346	33.0	359	34.3	0.000
	Elective	880	76.9	704	67.0	687	65.7	
								
**Region**	A	179	15.6	159	15.1	82	7.8	
	B	589	51.4	443	42.2	217	20.7	0.000
	C	227	19.8	273	26.0	558	53.3	
	D	150	13.1	175	16.7	189	18.1	
**Num. of secondary diagnoses**	Mean (sd)	2.9 (2.4)		3.7 (2.7)		4.7 (2.9)		0.000*
**Length of Stay (LOS)**	Median	16		21		21		0.000*

Total		1145		1050		1046		

*Kruskal-Wallis test.

In the regression model (table 5), increased age and urgent admission were independent risk factors for in-hospital mortality. Likewise, CHF and cardiac dysrhythmias were associated to an increased probability of dying in the hospital, while Billroth I and II interventions (partial gastrectomies with anastomosis to duodenum or jejunum), as well as simple lymphadenectomy were associated to a decreased probability of dying in the hospital. The Hosmer-Lemeshow statistic was 2.025 (p = 0.980) and the area under the ROC curve 0.772 (95%CI 0.747 – 0.797).

**Table 5:** Multivariate logistic regression model of in-hospital mortality.

	**p-value**	**OR**	**95%**	**CI OR**
			**Lower**	**Upper**

**Simple excision of lymphatic structure**	,005	,189	,058	,611
**Billroth I**	,001	,379	,212	,677
**Billroth II**	,002	,651	,496	,853
**Age**	,000			
**51–65**		3,237	1,359	7,714
**65–75**		4,383	1,885	10,191
**75–84**		8,266	3,569	19,141
**≥ 85**		13,913	5,598	34,574
**Type of admission: urgent**	,001	1,551	1,208	1,992
**Congestive Heart Failure**	,003	2,325	1,333	4,056
**Cardiac dysrhythmias**	,040	1,495	1,019	2,194
**Number of secondary diagnoses recorded**	,000			
**4–5**		3,410	2,031	5,724
**≥ 6**		8,691	5,154	14,656
**Hospital volume**	,242			
**18–35**		1,285	,949	1,741
**>35**		1,245	,892	1,736

Reference categories: simple excision of lymphatic structure (no); Billroth I (no); Billtroth II (no); age (≤ 50); type of admission (elective); number of secondary diagnosis (≤ 3); hospital volume (≤ 17); region (A). Adjusted by region.

OR: *Odds Ratio*

**Figure 1:**
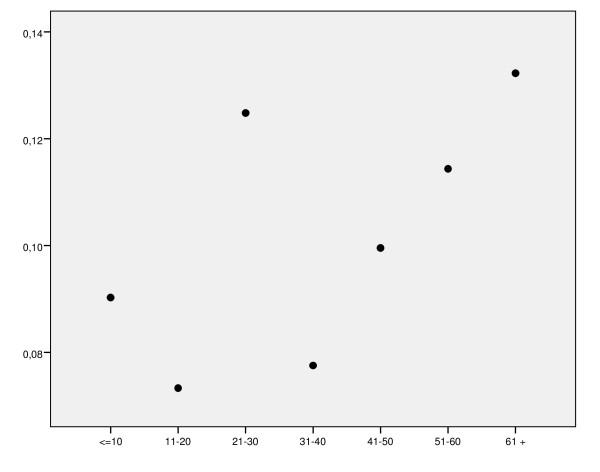
**In-hospital mortality rates of the centres grouped according to annual volume of discharges**.

Despite the association found between annual volume and crude in-hospital mortality, no specific pattern of crude in-hospital mortality was observed after grouping centres in smaller volume categories (see figure 1). In the logistic regression model, hospital volume grouped by terciles was not independently associated with mortality after adjusting for other factors.

**Figure 2:**
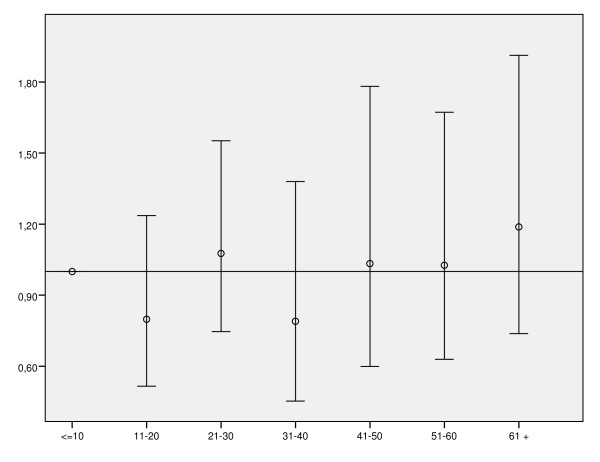
Variation in the Odds Ratios (95%CI) for adjusted* in-hospital mortality in relation to centres with lower volume (≤ 10 discharges). The circle indicates the estimated *Odds Ratio *(OR), while the vertical lines indicate the 95%CI of the OR. * Adjusted for age, type of admission, simple excision of lymphatic structure, Billroth I and Billroth II intervention, congestive heart failure, cardiac dysrhythmias, number of secondary diagnoses recorded, and region.

The *Odds Ratios *for in-hospital mortality, adjusted for the variables included in the regression model and using the smaller volume categories, are shown in figure 2. Again, we observed no trend or pattern that would enable a possible relation between volume and in-hospital mortality to be identified.

## Discussion

The in-hospital mortality rate in patients that underwent surgery for stomach cancer during 2001 and 2002 was greater than 10% in the overall set of regions evaluated. Older patient age, urgent admission, and certain comorbidities were significantly associated to greater mortality. Certain surgical procedures, such as Billroth I and II were associated to lower mortality. We found no relation between volume and in-hospital mortality.

### Comparison with past literature

Differences in study periods and the definition of mortality used (such as post-operative mortality, 30-day mortality, or in-hospital mortality) among the different studies published limits the comparability of results. Moreover, some studies, such as ours, did not adjust mortality rates for severity factors, such as tumour stage at diagnosis. Despite these limitations, we can say that the in-hospital mortality rate observed in our study was high, although it was within the range of 1.7% to 12% reported by other authors [2,21,22]. McCulloch et al. reported the exact same mortality rate in 4 years as found in our study [23]. Furthermore, the wide range of variability among hospitals in our study might be partly due to differences in the factors that we found were associated, as the estimations of the adjusted odds ratios for mortality at the different centres grouped according to volume (figure 2) are similar and their confidence intervals overlap.

### Hospital mortality and quality of care

Mortality has been defended as an indicator of the quality of care in hospitals. In fact, mortality is an objective, reliable, precise, and bias-free measure that can be the direct consequence of substandard care; however, a high mortality rate does not always indicate poor quality and poor quality does not always result in greater hospital mortality [24]. In the United States, the Agency for Healthcare Research and Quality (AHRQ) has approved the use of hospital mortality rates for 8 surgical procedures as criteria of quality and possible referral of patients to other centres [25]. These 8 procedures were selected because of their high mortality and because of the high variability in mortality among the different hospitals that they analyzed. Nevertheless, as Dimick et al. point out, the low frequency of some of these 8 surgical procedures at some centres raises the question whether it is appropriate to use mortality rates as a measure of quality in all cases [5].

### Study implications and limitations

From the information available in our study, it is difficult to deduce what aspects of the process of care (details about surgical management, for instance) have led to complications such as peritonitis, kidney failure, or respiratory failure, and this makes it difficult to take action to improve the quality of care. Likewise, suture failure can occur after technically impeccable surgery, because it depends to a certain extent on other factors such as the patient's nutritional and/or immune status. This is one limitation of hospital mortality studies that use administrative databases if the aim is to use the results to improve the process of care.

Furthermore, as some authors have already noted, administrative databases also have limitations for adjusting patients' baseline risks to enable comparisons of mortality rates [26-29]. These limitations are related to a) differences in (or the lack of) coding for some comorbidities or procedures and the consequent possibility of under-coding of diagnoses in patients with greater severity (for example, the variable relative to nutritional deficiencies), b) the misclassification of certain health problems, c) the failure to register some variables of known clinical importance (for example, the clinical stage of the tumour or indicators of the patient's pre-operative physiological state, which is of key importance in surgical patients [30,31]), and d) the difficulty in distinguishing among health problems that were present before admission from those that might have resulted from complications of the healthcare process.

We used in-hospital mortality as the outcome variable in the study because it was the only mortality variable available in the administrative databases; however, using this outcome variable can lead to limitations in interpreting differences in mortality. For instance, in-hospital mortality would probably be higher in centers that prolong LOS than in centers with a policy to discharge patients earlier. Patients discharged earlier might die within 30 days of discharge and this would result in an underestimation of surgical mortality. Table 4 shows that LOS was lower in centers with a lower volume of interventions, and this could partially explain the lower in-hospital mortality in those centers. As mentioned above, the different studies published use different definitions of mortality; Table 6 shows the most recent results about volume of interventions and short-term mortality for gastric cancer.

**Table 6:** Original studies about volume of interventions and short-term mortality in stomach cancer (published in English and indexed 1999–January 2008).

**REFERENCE**	**N**	**PERIOD**	**SOURCE**	**VOLUME CATEGORIES**	**MORTALITY** **TOTAL % and/or (range by volume)**	**DEFINITION**	**ADJUST**	**ASSOC**.
Bachmann MO, *Br J Surg*, 2002	405	1996–1997	CDB	Terciles	14	operative (≤ 30 days)	Yes	No
Birkmeyer JD, *Cancer*, 2006	9,403	2000–2002	ADB	Quintiles	7.3 – 10.1	operative (discharge or ≤ 30 days)	Yes	Yes
Birkmeyer JD, *N Eng J Med*, 2002	31,944	1994–1999	ADB	Quintiles	8.7 – 13.0	operative (discharge or ≤ 30 days)	No	Yes
Callahan MA, *Ann Surg*, 2003	6,434	1998–2001	ADB	Quartiles	8.4 (3.7 – 11.3)	in-hospital (discharge)	Yes	Yes
Damhuis RAM, *Eur J Surg Oncol*, 2002	1,978	1987–1997	R	<7, 7–10, >10	7.9 (3.1 – 15.1)	operative (≤ 30 days)	Yes	No
Finlayson EVA, *Arch Surg*, 2003	16,081	1995–1997	ADB	<9, 9–17, >17	6.9 – 8.7	in-hospital (discharge)	Yes	No
Gordon TA, *J Am Coll Surg*, 1999	705	1989–1997	ADB	≤ 10, 11–20... ≥ 201	10.9 (6.3 – 12.8) (total gastrectomy)	in-hospital (discharge)	Yes	No
Hannan EL, *Surgery*, 2002	3,711	1994–1997	ADB	Quartiles	6.2 (2.8 – 11.2)	in-hospital (discharge)	Yes	Yes
Hansson LE, *Eur J Surg*, 2000	1,024	1989–1995	CDB	Hospital level	12 (10 – 13)	in-hospital (discharge)	Yes	No
Jensen LS, *Scand J Surg*, 2007	537	1999–2004	ADB	<5, 5–20, >20	8.2 (8.0 – 8.4)	operative (discharge or ≤ 30 days)	No	No
Lin HC, *Ann Surg Oncol*, 2006	11,348	2000–2003	ADB	Quintiles	1.3 – 5.3	in-hospital (discharge)	Yes	Yes
McCulloch P, *Br Med J*, 2003	590	1999–2002	CDB	≤ 10, 11–20, ≥ 21	10.3	in-hospital (discharge)	Yes	Weak
Reid-Lombardo KM, *J Gastrointest Surg*, 2007	6,047	2001	R	Hospital level	5.5 – 9.9	operative (≤ 30 days)	Yes	Yes
Smith JK, *Arch Surg*, 2007	13,354	1998–2003	ADB	≤ 4, 5–10, ≥ 11	6 (4.9 – 6.8)	in-hospital (discharge)	Yes	Yes
Smith DL, *Ann Surg Oncol*, 2007	1,864	1999–2001	ADB	<3, 3–15, >15	0.8 – 9.5	in-hospital (discharge)	Yes	Yes
Thompson AM, *Br J Surg*, 2007	1,264	1997–1999	CDB	Quartiles	9.1 – 11.9	operative (discharge or ≤ 30 days)	No	No
Wainess RM, *J Gastrointest Surg*, 2003	23,690	1988–2000	ADB	Terciles	7.4 (6.5 – 8.3)	in-hospital (discharge)	Yes	No
Xirasagar S, *Eur J Surg Oncol*, 2008	6,909	1997–1999	ADB	Quartiles	18.5 – 24.5	6-month	Yes	No

CDB: Clinical database; ADB: Administrative database; R: Cancer register

According to our data, it is evident that the specific location of the tumour is also often under coded and that the mean number of secondary diagnoses registered varies among different hospital volumes. In our study, patients who died in hospital had a higher number of secondary diagnoses, and this confirms a certain register bias that favours patients who die in the hospital. On the other hand, the absence of tumour stage, a key factor for the patient's prognosis (especially long-term prognosis), is an evident limitation. We would expect only patients with the most advanced stages (although with the possibility of being cured by surgery, as in our study) to have a greater risk of in-hospital death after the intervention. Finally, some authors have advocated tumour resection with radical lymph-node excision (D2), claiming that long-term outcomes (survival) are better than with more conservative surgery (D1), although D2 also has greater post-operative morbidity and mortality that counteracts the possible benefits [32,33]. The coding system used for the MBDS-HD, the ICD9CM, does not allow us to distinguish between these specific aspects of the care process, not only because of possible under coding, but also because of the lack of specific codes for this or other procedures.

Many of the limitations of this study derive from the fact that compiling homogeneous, highly reliable, and specific information is impossible due to the lack of information systems and specific clinical registers for oncological surgery in Spain, and this problem obviously needs to be tackled. Otherwise, it will be practically impossible to completely analyze the process of care and outcomes for oncological surgery that will enable us to take measures to improve the quality of care.

Even with the possible limitations, we have been able to determine that mortality is greater for tumours located in the *fundus *or even in the *cardia*; many tumours of the *fundus *also invade the *cardia*. Also, we have observed a lower crude mortality following a Billroth I than after a Billroth II in tumours located outside the *cardia *or *fundus*. In cases in which a Billroth I was used, we assume that it was a small tumour and that the patient could benefit, leading to low in-hospital mortality.

Administrative databases like the one used in this study are currently the only source of information that is common to all centres, homogeneous, accessible, and considerably exhaustive; they contain epidemiological and clinical information about the hospital discharges for a given diagnosis in the Spanish healthcare system and in those of many other countries [34]. Moreover, in-hospital mortality is a highly reliable objective measure that is available in all of these administrative databases and that can be monitored over time. Therefore, these databases can serve as the starting part for the analysis of the quality of care and detection of possible problems that might have an impact on hospital mortality.

### Hospital volume and in-hospital mortality

We found no independent relation between adjusted hospital mortality and the volume of interventions at a hospital. Moreover, there does seem to be a similarity in the risk of in-hospital mortality and other indicators (LOS, Charlson score, urgent admission) among the centres that performed more than 17 interventions per year.

In a recent prospective study carried out in Scotland, Thompson et al. also found no relation between hospital volume and mortality after surgery for stomach cancer [35]. Two other studies carried out in the United Kingdom found a very weak relation favouring lower mortality with greater volume [23,36]. In our study, the univariate analysis found a statistically significant association between volume and mortality in the opposite direction to that hypothesized, so that greater volume was associated to greater mortality. This tendency was only observed in tumours located in the *cardia *or *fundus*, but it was not significant and may be due to the low number of cases. This possible association was not significant in the multivariate analysis, either; again, this could be due to the low number of *cardia *or *fundus *tumours and the association found between the type of procedure and mortality for tumours outside those locations.

In fact, hospitals that treat a larger number of cases might care for patients with more severe disease and more comorbidities who have a higher probability of complications, and this might predispose to a higher mortality rate. In our study, we identified a significantly higher percentage of patients with Charlson score greater than or equal to 3 (see table 4) in higher volume centres, and a higher rate of complications of surgical procedures in those centres (data not shown).

However, some studies of mortality after surgery for stomach cancer have found that university hospitals and those that treat a greater volume of cases might have lower post-operative and long-term mortality rates, [10,37-41] although the results of other studies contradict this hypothesis or fail to confirm it [3,4,21,35,42-44] and other authors have questioned this hypothesis with respect to surgery for other types of cancer [45].

Despite the limitations in the comparability of the studies, it seems clear that the results are contradictory and there is no consensus, as is shown in table 6. It is evident that the characteristics of the healthcare systems, such as patient referral practices, centralization of oncological surgery, and the financing of medical procedures varies widely between the United States, Japan, Canada, and most European countries in which these questions have been analyzed. In the Spanish National Healthcare System, all medical procedures are financed by the System and patients are free to choose the centre where they are treated. On the other hand, the most complex patients tend to be treated in the centres that have the most experience in this type of interventions, so there is a certain degree of centralization Differences in the degree of centralization might partially explain the disparity in the cut-off points used for the volume of interventions in the different studies. Greater centralization can also lead to two different effects. On the one hand, centres specializing in a certain intervention are likely to attract more complex or more severe patients with greater a priori possibilities to die in the short term; on the other hand, increased volume and greater experience are likely to counterbalance these effects on in-hospital mortality.

Thus, it seems logical that any interpretation of the results published should take into account not only the comparability of the design of the studies, but also the specific characteristics of each healthcare system and the time frame of the observations [46]. Furthermore, it might be a mistake to consider greater volume as a standard to predict better quality, when it is more likely the structures, the experience and specialization of the professionals, and the many different processes linked to this type of intervention that are responsible for better results, as many authors have pointed out [47-49].

## Conclusion

In conclusion, this is the first study to be carried out in Spain that used secondary databases to analyze hospital mortality and possible associated factors after surgery for gastric cancer. Our results corroborate the existence of patient and intervention factors associated to greater hospital mortality, although we have found no clear association between the volume of cases treated at a centre and hospital mortality.

## Competing interests

The authors declare that they have no competing interests.

## Authors' contributions

MB conceived and directed the study, guided the statistical analysis, and drafted the manuscript. JC answered the questionnaire for specialists, participated in the design and the discussion of the results, and reviewed the manuscript. JR prepared the final database, performed the statistical analysis and participated in the discussion of the results. GN participated in the discussion of the results and reviewed the draft manuscript. CP and RC answered the questionnaire for specialists and reviewed the manuscript. AS participated in the design of the study. All authors read and approved the final manuscript.

## Appendix 1

Clinical Classification Software – DIAGNOSES

(With all coding changes valid from January 1980 through September 2003)

*Important: complications (not included in the multivariate model) are written in bold face*

Pneumonia (except that caused by tuberculosis or sexually transmitted disease)

00322 0203 0204 0205 0212 0221 0310 0391 0521 0551 0730 0830 1124 1140 1144 1145 11505 11515 11595 1304 1363 4800 4801 4802 4808 4809 481 4820 4821 4822 4823 48230 48231 48232 48239 4824 48240 48241 48249 4828 48281 48282 48283 48284 48289 4829 483 4830 4831 4838 4841 4843 4845 4846 4847 4848 485 486 5130 5171

Urinary tract infections

03284 59000 59001 59010 59011 5902 5903 59080 59081 5909 5950 5951 5952 5953 5954 59581 59582 59589 5959 5970 59780 59781 59789 59800 59801 5990

Peritonitis and intestinal abscess

03283 5670 5671 5672 5678 5679 5695

Complications of surgical procedures or medical care

3490 3491 41511 4294 4582 5121 5190 51900 51901 51902 51909 53640 53641 53642 53649 56962 5642 5643 5644 5696 56962 5793 9093 9954 99586 9970 99700 99701 99702 99709 9971 9972 9973 9974 9975 99760 99761 99762 99769 99771 99772 99779 9979 99791 99799 9980 9981 99811 99812 99813 9982 9983 99832 9984 9985 99851 99859 9986 9987 9988 99881 99882 99883 99889 9989 9990 9991 9992 9993 9994 9995 9996 9997 9998 9999

Intestinal obstruction without hernia

5600 5601 5602 56030 56031 56039 56081 56089 5609

Renal failure

5845 5846 5847 5848 5849 586 V420 V451 V560 V561 V562 V5631 V5632 V568 585 7925

Phlebitis, thrombophlebitis and thromboembolism

V1251 V1252 4510 45111 45119 4512 45181 45182 45183 45184 45189 4519 452 4530 4531 4532 4533 4538 4539

Respiratory failure, insufficiency, arrest (adult)

V461 V462 5185 51881 51882 51883 51884 7991

Acute myocardial infarction

4100 41000 41001 41002 4101 41010 41011 41012 4102 41020 41021 41022 4103 41030 41031 41032 4104 41040 41041 41042 4105 41050 41051 41052 4106 41060 41061 41062 4107 41070 41071 41072 4108 41080 41081 41082 4109 41090 41091 41092

Pancreatic disorders (not diabetes)

5770 5771 5772 5778 5779 5794

Hypertension

4011 4019 4010 40200 40201 40210 40211 40290 40291 4030 40300 40301 4031 40310 40311 4039 40390 40391 4040 40400 40401 40402 40403 4041 40410 40411 40412 40413 4049 40490 40491 40492 40493 40501 40509 40511 40519 40591 40599 4372

Diabetes mellitus with complications

25002 25003 25010 25011 25012 25013 25020 25021 25022 25023 25030 25031 25032 25033 25040 25041 25042 25043 25050 25051 25052 25053 25060 25061 25062 25063 25070 25071 25072 25073 25080 25081 25082 25083 25090 25091 25092 25093

Nutritional deficiencies

V121 260 261 262 2630 2631 2632 2638 2639 2640 2641 2642 2643 2644 2645 2646 2647 2648 2649 2650 2651 2652 2660 2661 2662 2669 267 2680 2681 2682 2689 2690 2691 2692 2693 2698 2699 7994

Gastrointestinal haemorrhage

4560 45620 5307 53082 53100 53101 53120 53121 53140 53141 53160 53161 53200 53201 53220 53221 53240 53241 53260 53261 53300 53301 53320 53321 53340 53341 53360 53361 53400 53401 53420 53421 53440 53441 53460 53461 5693 5780 5781 5789

Invasion of other structures

1974 1975 1976 1977 1978

Diverticulosis and diverticulitis

56200 56201 56202 56203 56210 56211 56212 56213

Other gastrointestinal disorders

V127 V1270 V1279 V416 V441 V442 V443 V444 V453 V473 V535 V551V552 V553 V554 5581 5582 5640 56400 56401 56402 56409 5641 5645 5647 5648 56481 56489 5649 5680 56881 56882 56889 5689 56981 56982

56983 56984 56985 56986 56989 5699 5790 5791 5792 5798 5799 7871 7872 7873 7874 7875 7876 7877 7879 78791 78799 7892 7893 78930 78931 78932 78933 78934 78935 78936 78937 78939 7894 78940 78941 78942

78943 78944 78945 78946 78947 78949 7899 7921 7934 7936

Fluid and electrolyte disorders

2760 2761 2762 2763 2764 2765 2766 2767 2768 2769

Cardiac dysrhythmias

4270 4271 4272 42731 42732 42760 42761 42769 42781 42789 4279 7850 7851

Congestive heart failure, no hypertensive

39891 4280 4281 42820 42821 42822 42823 42830 42831 42832 42833 42840

42841 42842 42843 4289

Malignant neoplasm of stomach:

1510 Cardia

1511 Pylorus

1512 Pyloric antrum

1513 Fundus of stomach

1514 Body of stomach

1519 Stomach, unspecified

Operations on lymphatic system

40.2 Simple excision of lymphatic structure

40.3 Regional lymph node excision

40.5 Radical excision of other lymph nodes

Incision and excision of stomach

43.5 Partial gastrectomy with anastomosis to oesophagus (proximal gastrectomy)

43.6 Partial gastrectomy with anastomosis to duodenum (Billroth I)

43.7 Partial gastrectomy with anastomosis to jejunum (Billroth II)

43.8 Other partial gastrectomy

43.9 Total gastrectomy

## Pre-publication history

The pre-publication history for this paper can be accessed here:


